# Convergence between helminths and breast cancer: intratumoral injection of the excretory/secretory antigens of the human parasite *Toxocara canis* (EST) increase lung macro and micro metastasis

**DOI:** 10.3389/fimmu.2024.1332933

**Published:** 2024-03-20

**Authors:** Raúl Aragón-Franco, Rocío Alejandra Ruiz-Manzano, Karen Elizabeth Nava-Castro, Víctor Hugo Del Rìo Araiza, Claudia Angelica Garay-Canales, Armando Pérez-Torres, Romel Chacón-Salinas, Manuel Iván Girón-Pérez, Jorge Morales-Montor

**Affiliations:** ^1^ Departamento de Inmunología, Escuela Nacional de Ciencias Biológicas, Instituto Politécnico Nacional (ENCB-IPN), Ciudad de México, Mexico; ^2^ Laboratorio de Neuroinmunoendocrinología, Departamento de Inmunología, Instituto de Investigaciones Biomédicas, Universidad Nacional Autónoma de México, Ciudad de México, Mexico; ^3^ Laboratorio de Biología y Química Atmosférica, Departamento de Ciencias Ambientales, Instituto de Ciencias de la Atmósfera y Cambio Climático, Universidad Nacional Autónoma de México, Ciudad de México, Mexico; ^4^ Laboratorio de Interacciones Endocrinoinmunitarias en Enfermedades Parasitarias, Facultad de Medicina Veterinaria y Zootecnia, Departamento de Parasitología, Universidad Nacional Autónoma de México, Ciudad de México, Mexico; ^5^ Departamento de Biologia Celular y Tisular, Facultad de Medicina, Universidad Nacional Autónoma de México, Ciudad de México, Mexico; ^6^ Laboratorio de Inmunotoxicología, Secretaría de Investigación y Posgrado, Universidad Autónoma de Nayarit, Tepic, Nayarit, Mexico

**Keywords:** breast cancer, tumor microenvironment, *Toxocara cannis* excretory/secretory antigens, metastasis, risk factors

## Abstract

**Introduction:**

Worldwide, breast cancer is the most important cancer in incidence and prevalence in women. Different risk factors interact to increase the probability of developing it. Biological agents such as helminth parasites, particularly their excretory/secretory antigens, may play a significant role in tumor development. Helminths and their antigens have been recognized as inducers or promoters of cancer due to their ability to regulate the host’s immune response. Previously in our laboratory, we demonstrated that chronic infection by *Toxocara canis* increases the size of mammary tumors, affecting the systemic response to the parasite. However, the parasite does not invade the tumor, and we decided to study if the excretion/secretion of antigens from *Toxocara canis* (EST) can affect the progression of mammary tumors or the pathophysiology of cancer which is metastasis. Thus, this study aimed to determine whether excretion/secretion *T. canis* antigens, injected directly into the tumor, affect tumor growth and metastasis.

**Methods:**

We evaluated these parameters through the monitoring of the intra-tumoral immune response.

**Results:**

Mice injected intratumorally with EST did not show changes in the size and weight of the tumors; although the tumors showed an increased microvasculature, they did develop increased micro and macro-metastasis in the lung. The analysis of the immune tumor microenvironment revealed that EST antigens did not modulate the proportion of immune cells in the tumor, spleen, or peripheral lymph nodes. Macroscopic and microscopic analyses of the lungs showed increased metastasis in the EST-treated animals compared to controls, accompanied by an increase in VEGF systemic levels.

**Discussion:**

Thus, these findings showed that intra-tumoral injection of *T. canis* EST antigens promote lung metastasis through modulation of the tumor immune microenvironment.

## 1 Introduction

Cancer is one of the main causes of mortality worldwide and is considered a public health problem. Particularly breast cancer is the most important in terms of prevalence in women, in addition to the fact that it is the one with the highest incidence in women in 154 countries (1). The progression of breast cancer includes three phases: benign, non-invasive, and invasive. In the benign and non-invasive phases, transformed cells do not cross the basement membrane, and these stages are differentiated purely by histological grade (2). In invasive cancer or the metastasis stage, tumor cells migrate through the basement membrane to surrounding tissue and other organs (3). Immune cells detect and destroy transformed cells in a phenomenon called immunosurveillance. The tumor can also remain in a state of dormancy (immunoequilibrium) because of the control of immune cells and immunosurveillance (4). But, when transformed cells evade these elimination mechanisms (immunoscape), they survive and proliferate.

In the tumor microenvironment, non-neoplastic cells (fibroblasts, endothelial cells, mesenchymal cells, innate and adaptive immune cells) interact with tumor cells to form a dynamic tumor microenvironment; when the balance between the interactions of stromal cells, immune cells, and tumors, along with their secretion products, is lost, local “immunosuppression” occurs, and non-neoplastic cells acquire a “pro-tumor” phenotype which leads to tumor development (5, 6). The angiogenic process has been linked to the ingrowth of new blood vessels to increase oxygen and nutrient delivery to the tumor, allowing tumor cells to proliferate (7). This is supported by elevated VEGF levels in triple-negative breast tumors (TNBCs), which are correlated with a worse prognosis, recurrence, and patient survival (8). Furthermore, in breast cancer, there is a correlation between large numbers of infiltrated Treg lymphocytes, a more advanced progression of the tumor, and a high-risk and late relapse prognosis (9–11).

On the other hand, there are many risk factors associated with breast tumor progression. The first and most important is sex; women acquire the disease much more than men. Other risk factors are early menarche, first childbirth after 30 years of age, late menopause, nulliparity, overweight or obesity, personal or family history of breast cancer, alcoholism, smoking, menopausal hormone replacement therapy, and the use of hormonal contraceptives (12–14); also chemical, physical, and biological environmental contaminants represent risk factors for the development of this type of cancer (4). Within the latter are infectious agents that include viruses, fungi, bacteria, and parasites (15). Although biological agents are ubiquitous, the relationship between their presence and the development of different types of cancer has been moderately addressed, even though it has been reported that 15% of different types of cancer are linked to infections by viruses, bacteria, or parasites (16). Infectious agents of medical importance imply a health problem not only because of their impact-inducing disease but also because they have been associated with cancer due to the activation of chronic immunological processes in their host (15).

Among the parasites associated with cancer, helminths play an important role in tumor progression, due to their ability to modify the host’s immune response and their ubiquity in the environment. Different flukes are described as carcinogenic for bladder cancer (*Schistosoma haematobium*) and cholangiosarcoma (*Chlonorchis sinensis and Opisthorchis viverrini*) (17, 18). In this sense, different mechanisms have been described by which helminths promote tumor progression. Most are associated with chronic infections, which these parasites usually produce and mediate through excretion and secretion products (ES). Chronic inflammation leads to genomic instability and mutations, downregulation of suppressor genes, inhibition of apoptosis, and modulation of the immune response, which is why it can benefit tumor progression (17, 18). The importance of infection by helminths is not only related to their regulatory functions but also to their ubiquity and geographic distribution. Recently, focus has been placed on their ability to induce co-morbidities, due to excretion/secretion products within the host.

A nematode holding these immunomodulatory characteristics that infect humans and other hosts worldwide is *Toxocara canis* (Werner, 1782) (19). *Toxocara canis* infection is a cosmopolitan, zoonotic, and neglected disease. Domestic and wild canids, including dogs, are its definitive hosts, mainly affecting puppies under three months of age due to perinatal transmission (20, 21). In humans, infection by *T. canis* is a prevalent and neglected disease, even in industrialized countries (22).

The biological cycle of *T. canis* is complex and varies depending on the host type (definitive or paratenic), host`s specie (mouse, rat, dog, human) host’s age (pup or adult), and physiological state (gestating or non-gestating). For instance, in pregnant bitches, somatic larvae go through a reactivation process consisting of the migration of larvae toward the uterus and mammary glands.

During *T. canis* infection, the predominant immune response is a Th2 type, although a dichotomy has also been mentioned in terms of the polarization of the immune response. It is possible to find molecules of both the Th1 and/or Th2 type. This response may be influenced by aspects such as the host species (dog, cat, mouse, rat, human), time of infection (acute or chronic), and target organ (lung, liver, brain), among others. In general, the immune response is assembled against somatic antigens and excretory-secretory antigens (TcES-Ag) (17–19). This response is characterized by a strong adaptive immune response, where a Th2-type response predominates during the chronic infection. The Th2 response is characterized by the production of cytokines such as interleukin (IL) IL-4, IL-5, IL-6, IL-13, IL-33, and regulatory cytokines such as IL-10, among others (17–21). However, during the acute phase of infection, a strong pro-inflammatory response takes part in the immunological profile, where a mixed response with inflammatory cytokines of innate origin, together with Th17 and Th2 type cytokines has been reported (22).

As mentioned, both the survival and pathogenic action of *T. canis* larvae on their hosts are mediated by their excretion and secretion products (EST). These promote and modulate the host’s immune response while evading it so that the parasite can live, even for years, in the host’s tissues (19). In the *in vitro* culture supernatant of T lymphocytes obtained from human peripheral blood cells of *T. canis*-positive donors, stimulated with EST, increased levels of IL-4 and IL-5 were detected (23). Considering that *T. canis* can potentially induce a regulatory response in its hosts, it is important to consider the association with other diseases that may be favored by a tolerant environment, such as cancer. Therefore, this work aimed to characterize the intratumoral immune response of the host to ES antigens of *T. canis* and subsequently analyze its interaction with the progression to metastasis. Breast cancer, which is the most important in terms of incidence and prevalence in women, can be favored by the induction of a regulatory immune microenvironment. Therefore, it is necessary to evaluate the influence of the excretion/secretion products of *T. canis* on the growth of mammary tumors.

## 2 Materials and methods

### 2.1 Ethical statement

Experimental and animal care procedures were performed at the Institute of Biomedical Research (IIB), National Autonomous University of Mexico (UNAM) in the Biological Models Unit (UMB). The Institutional Committee for Care in the Use of Laboratory Animals (CICUAL) evaluated and approved the experimental procedures (permit number 2017-208), in adherence to the Mexican standard (NOM-062-ZOO-1999) and the National Institute of Health (NIH) Guide for Care and Use of Laboratory Animals. All efforts were made to minimize the number of animals used and their suffering.

### 2.2 Animals

For the experiments, female mice of the syngeneic BALB/c strain were kept at the UMB, UNAM. under the following standard conditions: controlled temperature of 22°C and 12 hours of light and dark cycles. Purina LabDiet 5015 food and water (Purina, St. Louis MO) were administered, both under sterile conditions. Blood was collected through cardiac puncture from mice deeply anesthetized with 5% v/v Sevoflurane (Abbot). Subsequently, the euthanasia of the animals was performed through cervical dislocation. Serum samples were obtained by centrifugation of blood (3250xg / 10 min, HERMLE Z400K) and stored at -70°C until use.

### 2.3 Obtaining and quantification of excretion and secretion products from *T. canis* (EST)

The *T. canis* larvae excretion and secretion products (EST) were obtained based on the methodologies described by De Savigny and Bowman, with modifications. Briefly, the larval egg suspension was centrifuged (3250 g/5 min), and the pellet was resuspended with 1 ml of 1% sodium hypochlorite to break the outer membrane of the eggs, which was stimulated by gently shaking for no more than 10 minutes. The eggs were transferred to a 15 ml centrifuge tube with 10 ml of double distilled sterile water and centrifuged at 3,250xg / 5 min; later, PBS was added for three additional washes. The pellet was resuspended in 1 ml of RPMI 1640 culture medium without phenol red (Sigma, St. Louis MO) with 1% Antibiotic-Antimycotic (GIBCO). The larval suspension was incubated with 20 ml of RPMI medium under mild agitation for 20 min with a magnetic stirrer to promote larval hatching. Subsequently, the larval suspension was incubated overnight at 37°C, in an atmosphere containing 5% CO2 (v/v).

Using a modified Baerman apparatus, larvae were separated from shells, larval eggs, and debris. The larvae were maintained in 20 ml of RPMI 1640 medium, at 37°C with 5% CO2, at a concentration of 104 larvae/ml. Every week, 10 ml of the supernatant from the larvae culture was collected and replaced with a fresh medium. The collected medium was filtered through a 0.2 µm syringe filter (Millipore) and precipitated with 1:8 dilution of acetone (Herschi Trading, High Purity, 99.5%) at -20°C overnight; subsequently, centrifuged at 8450 g/4°C/15 minutes. The pellets were pooled and allowed to dry in sterile conditions. Later, 50-100 µl of PBS were added, and the samples were stored at -20°C until use. The protein concentration was determined with a Bradford Protein Assay Bio Rad^®^ using six serial dilutions to build the standards curve with albumin.

### 2.4 Cell culture

Mammary mouse carcinoma cell line 4T1 (ATCC^®^ CRL-2539) was kindly donated by Dr. Karen Nava Castro. 4T1 cells were thawed and washed with 10 ml of RPMI 1640 (Sigma, St. Louis, MO) without supplementation, then centrifuged at 791 g/5 min. The pellet was resuspended with RPMI 1640 medium (Sigma, St. Louis, MO) supplemented with 10% fetal calf serum (ByProducts, Guadalajara, Mexico), 1.0 mM sodium pyruvate, 100 U/ml penicillin, and 100 mg/ml streptomycin. Cells were incubated at 37°C, 5% CO2 (v/v) in a sterile flask. A passage was carried out once the monolayer of adherent cells reached 80% confluence. After three PBS washes, the cells were detached with 0.25% trypsin/EDTA (Sigma Aldrich) and resuspended in a supplemented culture medium. After two passages, the cells were harvested for inoculation. The cells were detached as described above, and 500 µl of FBS and 500 µl of PBS were added. Subsequently, the cells were centrifuged at 791 g/5 min, and the pellet was washed with sterile NaCl 0.9% isotonic saline solution (ISS 0.9%). We determined the number of cells with a Neubauer chamber, using trypan blue to check viability.

### 2.5 Tumor induction

After a second subculture at 80% of confluency, 4T1 cells were harvested and suspended in sterile 0.9% NaCl solution at a concentration of 250,000 cells/mL. They were conserved in ice until the injection into the mice. Upon sexual maturity (8 weeks old), mice of every exposure group were randomized into secondary experimental groups, i.e. Control (without tumor induction) and 4T1 (tumor induction) groups. For the mammary tumor induction, mice were anesthetized by inhalation of a mixture of air and 5% sevofluorane. (Abbot, Mexico), and the abdominal area was cleaned with EtOH 70%. Mice recovery was supervised. Mice assigned to 4T1 groups were treated as follows. After low abdomen asepsis was performed and the 4th nipple was located, 10^4^ cells of 4T1 cell line were injected into the mammary fat pad. Tumor growth was monitored daily during 25 days.

### 2.6 Intratumoral EST treatment

To test the intratumoral effect of ESTs on tumor growth, female mice were randomized into the following experimental groups: 1) Intact group of animals; 2) 4T1 group of animals with untreated tumors; 3) ESTs group of mice with intratumoral injection of 1160 ng in 40 µL of PBS. Tumor growth was observed for 28 days. Finally, the tumor weight was obtained at the moment of the euthanasia on day 28 post-inoculation.

### 2.7 Histological analysis of lungs

According to the Mexican Official Guide, all mice were euthanized by isoflurane overdoses. To prevent alveolar collapse, whole lungs were fixed through intratracheal perfusion with 500 µL of 4% paraformaldehyde diluted in isotonic saline solution. After thoracotomy, lungs were dissected and then submerged in the same fixative solution for a minimum of 24 h. After, the lungs were rinsed in tap water, dehydrated through ascending ethanol grades, cleared in xylene, and embedded in paraffin with an orientation to obtain transversal sections from both lungs. Sixteen lung histological sections from each group of mice (4 μm thick and separated from each other by 100 μm), were stained with hematoxylin-eosin and analyzed using a BX50 Olympus microscope equipped with a digital camera and the Infinity Analyze software, v6.3.0.

### 2.8 Spleen and tumor protein extraction

Spleen and tumor from mice were obtained and stored in TRIzol™ (Ambion) at -70°C until use. Protein separation was performed using the TRIzolTM (Ambion) procedure guidelines with modifications. Briefly, the tissue was homogenized in TRizol with the Polytron (Kinematica), and chloroform was added and shaken for 20 minutes. Subsequently, samples were incubated for 2-3 min and centrifuged (8450 g/4°C). The organic phase was separated, and 100% EtOH was added. Each tube was vortexed briefly, incubated for 2-3 min, and centrifuged (8450xg / 4°C). Cold isopropanol was added and incubated for 10 min; the pellet was washed three times with guanidinium thiocyanate (0.3 M). 100% EtOH was added again, centrifuged, and allowed to dry. The proteins were solubilized with 0.01% sodium dodecyl sulfate (SDS) and quantified with the NanoDrop 1000 spectrophotometer (Thermo-Scientific). 10 µg of protein was used to determine tissue cytokine levels.

### 2.9 Flow cytometry

A sample of breast tumors was collected with a scalpel and placed in 500 µl of digestion solution (Appendix 8.1.11). The reaction was stopped with 50 µl of Fetal Bovine Serum (FBS) and disintegrated between two nylon meshes (50 µm pore diameter). The cell suspension was collected, centrifuged at 1250 rpm/5 min/4°C, and resuspended in 200 µl of FACS buffer. Approximately 1x106 cells were incubated (20 min at 4°C) with anti-CD16/CD32 (TruStain^®^, BioLegend, San Diego CA) and washed with FACS buffer. The FoxP3/Transcription Factor Staining Buffer kit (Tonbo Biosciences, San Diego CA) was used for intracellular staining of FoxP3, according to the supplier’s protocol. After cell permeabilization, intracellular staining with anti-FoxP3 was performed for the detection of Treg cells.

Panels of NK cells/macrophages and B cells were fixed with 100 µl 4% paraformaldehyde/PBS and 100 µl FACS buffer for 20 min, centrifuged, decanted, and resuspended in 200 µl FACS buffer. All labeled antibodies used for flow cytometry were from BioLegend (San Diego CA, USA). Compensation controls of individual stainings were performed for all four fluorochromes and all three tissues. Cellular analysis was performed using the FACSCalibur™ flow cytometer (BD Biosciences). Data was analyzed with the BD CellQuest™ program. Compensation was performed on the FACSCalibur™ cytometer and FlowJo software (Becton, Dickinson and Company; 2023), using unstained samples, single stains as controls, and FMO for Foxp3 (CD3+/CD4+; CD3+/CD8+).

### 2.10 Selection of immune populations in tumor, spleen, and peripheral lymph nodes

**Figure 1:**
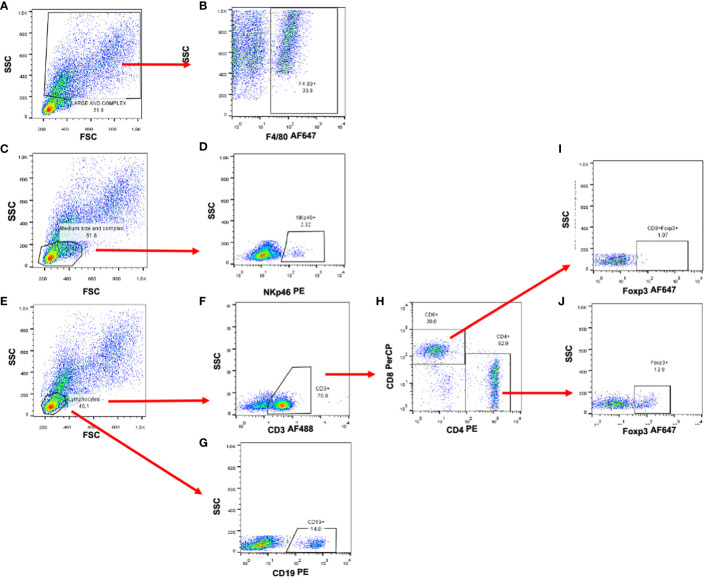
Selection strategy for the identificaction of immune cell subpopulations. Representative dot plots od murine splenocytes. **(A, C, E)** Forward and side scatter plots used to identify cell populations by size and complexity. **(B)** Macrophage identification by the expression of F4/80 marker. **(D)** NK cell selection using NKp46 marker. **(F)** Total T cells were selected by the expression of CD3épsilon and then **(H)** Th and Tc cells by the expression of CD4 and CD8 molecules. **(I, J)** Regulatory T cells were analyzed and selected by the expression of the Foxp3 transcription factor. **(G)** B cells were selected by the expression of CD19 cell marker.

To discriminate the different populations of immune cells located in the tumor or immune organs such as the spleen or peripheral lymph nodes, we used the following strategy: cells were selected by size and granularity through forward versus side scatter (FSC vs SSC) gating. From this gate (FSC vs SSC), the F4/80+ cells were chosen from the largest and most complex cells (**Figures 1A, B**). Lymphocytic subpopulations were selected in the small and no complex cells (**Figures 1C, E**). NK cells were then selected by the expression of NKp46 (**Figures 1C, D**). For T cells, a subpopulation of CD3+ cells were selected (**Figure 1F**) and then, by the expression of CD4 and CD8 markers (**Figure 1H**). Regulatory T cells, were identified by the expression of FOXP3 (**Figures 1I, J**). Finally, B cells were identified by the expression of CD19 (**Figure 1G**).

### 2.11 Sandwich ELISA for the detection of cytokines

Serum and splenic cytokines were measured with the ABTS ELISA kits (PeproTech) according to the Peprotech protocol indications. The following antibodies were used: TNF-α (500-P64), IFN-γ (500-P119), IL-4 (500-P54), IL-5 (500-P55), and IL-10 (500-P60), using capture and detection antibodies, to prepare the sandwich ELISA. The enzyme-substrate reaction was developed with the ABTS substrate (PeproTech), and the plates were read at a wavelength of 405 nm with a correction of 650 nm, at different reading times, in the Stat Fax 4200 microplate reader (Awareness Technology).

### 2.12 Indirect ELISA for VEGF quantification

Polystyrene 96-well plates were sensitized (Maxisorp Nunc) with 50 µl of protein (10 µg), serum (1:2 dilution), or VEGF (Santa Cruz Biotechnology) standard (0.001 ng-1ng) in bicarbonate buffer (in duplicate) and incubated at 4°C overnight following manufacturer instructions. Subsequently, the plates were washed three times with a wash solution. 200 µl of the blocking solution (1%) was added and incubated for one hour at 4°C. Next, 50 µl of anti-VEGF C-1 antibody (sc-7269, Santa Cruz Biotechnology) (1:200) was incubated for one hour at 4°C. For recognition of anti-VEGF IgG, 50 µl of m-IgGκ BP-HRP (sc-516102 Santa Cruz Biotechnology) was added (1:400) for 2 h at room temperature. Finally, after washing, the enzyme-substrate reaction was performed with 50 µl of the chromogen solution. The reaction was stopped after 20 min with 50 µl of 2 N sulfuric acid. The absorbances were determined at 492 nm in the Stat Fax 4200 microplate reader (Awareness Technology). All cytokine and VEGF concentrations were calculated with interpolation from a standard curve. The determination of TES concentration, dilutions of sera, and antibodies was developed after the standardization of the respective ELISAs.

### 2.13 Statistical analysis

The data obtained from the experiments were plotted as the mean ± SD. To analyze the differences between the mice exposed to excretion and secretion products (TES) and control animals, we used an ANOVA test, followed by a Tukey test. In all cases, the differences were considered significant when p ≤ 0.05. Linear regression and correlation were performed to determine if the values of the different methods were close to the actual volume of the tumors. All analyses were calculated using Prism 8 ^®^ software (GraphPad Software Inc.).

## 3 Results

### 3.1 Injection of EST into the tumor does not significantly increase the tumor size

**Figure 2:**
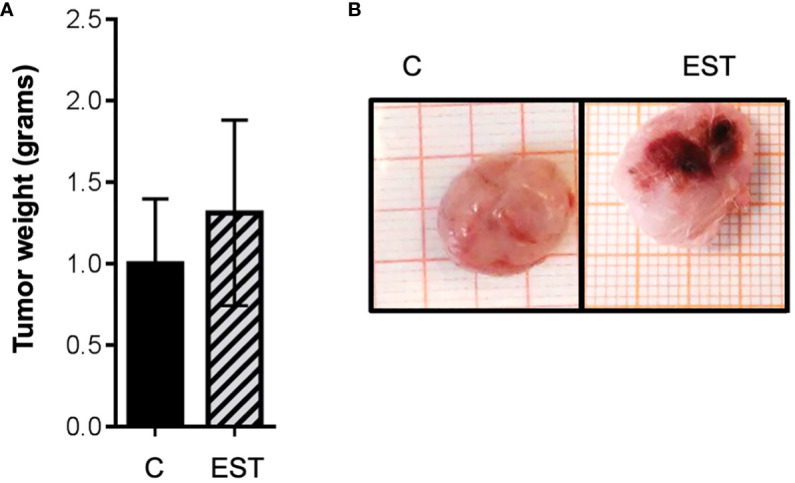
Weight and size of the tumor masses with intratumoral injection of EST. **(A)** Average weight of the tumors is shown, and **(B)** the demonstrative photographs of the intact groups and those that were injected intratumorally, the vehicle (C), and the secretion and excretion antigens (EST). The bars represent the average ± SD (Vh, n = 5; EST, n= 8).

Since the host’s immune response to *T. canis* larvae is mediated by their excretion and secretion products (EST), we included groups of animals injected with EST intratumorally or injected only with the vehicle PBS (C). Although visually the tumors appeared larger (**Figure 2B**), no significant differences were obtained between the weight of the tumors of the groups of animals injected with EST compared with vehicle (C) (**Figure 2A**). However, a very revealing finding, it is the fact that those tumors from the EST group are much more vascularized than controls and vehicle. The later indicates that progression to metastasis is facilitated by the presence of these antigens (EST) (**Figure 2B**).

### 3.2 Intratumoral injection of EST does not modify the immune tumor microenvironment

Tumors are very heterogeneous and consist of many different cells, including of course immune cells, in addition to the tumor cells. Interactions of all these cells results in primary tumor proliferation and, as a result, high vascularization and metastasis. These cellular and peripheral signals are referred to as the Tumor Microenvironment (TME), consisting of immune cells, endothelial cells, adipocytes, cancer-associated fibroblasts (CAFs), and non-cellular substances such as ECM and cytokines (24). This mixture of components of TME impacts the outcome of the patient since it affects cancer initiation, progression, metastases, and treatment resistance (25).

**Figure 3:**
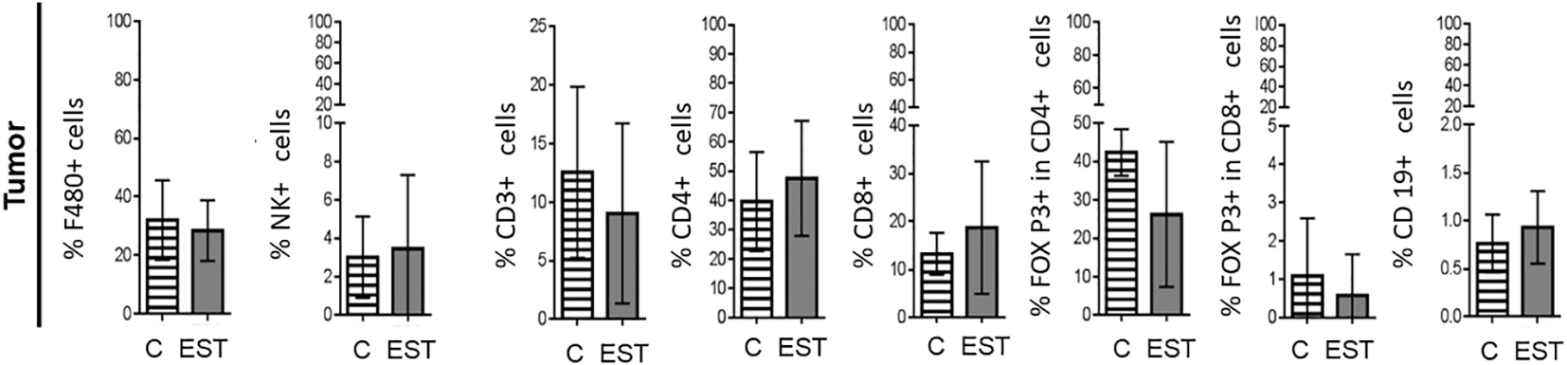
Subpopulations of cells in tumors from animals injected intratumorally with EST. Determination of the frequencies of Macrophages (F4/80+), Natural killer (NK), T Lymphocytes (CD3+), helper T Lymphocytes (CD4+), cytotoxic T lymphocytes (CD8+), Treg CD4+, Treg CD8+ and B (CD19+) lymphocytes in the tumor. The bars represent the average ± SD (Vh, n = 5; EST, n= 8).

On the other hand, secretion and excretion products (EST) from *T. canis* stimulate inflammation which promotes recruitment, polarization, or differentiation of immune cells to control the infection; in addition, this inflammation could promote tumor progression by different means. Based on the above, first, we decided to determine if mice intratumorally administered with EST modulate the immune cell composition of the TME compared to mice injected only with the vehicle (C). After enzymatic digestion of tumor, the released cells were labeled as indicated above. In none of the subpopulations measured significant differences were found between cell proportions: Macrophages (F4/80+), Natural killer (NK), T Lymphocytes (CD3+), helper T Lymphocytes (CD4+), cytotoxic T lymphocytes (CD8+), Treg CD4+, Treg CD8+ and B (CD19+) lymphocytes within the tumor analyzed (**Figure 3**).

### 3.3 Intratumoral injection of EST does not modify the systemic immune response in the spleen

**Figure 4:**
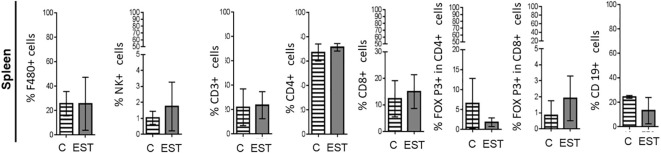
Subpopulations of cells in the spleen of animals injected intratumorally with EST. Determination of the frequencies of Macrophages (F4/80+), Natural killer (NK), T Lymphocytes (CD3+), helper T Lymphocytes (CD4+), cytotoxic T lymphocytes (CD8+), Treg CD4+, Treg CD8+ and B (CD19+) lymphocytes). The bars represent the average ± SD (Vh, n = 5; EST, n= 8).

Since a representative composition of immune cells is located in the spleen, and partially reflects the immune response, we proceeded to determine the proportion of immune cells. We performed measurements of the immune subpopulations in the spleen of the animals injected. No significant differences were found between cell proportions: Macrophages (F4/80+), Natural killer (NK), T Lymphocytes (CD3+), helper T Lymphocytes (CD4+), cytotoxic T lymphocytes (CD8+), Treg CD4+, Treg CD8+ and B (CD19+) lymphocytes within the analyzed spleen (**Figure 4**).

### 3.4 Intratumoral injection of EST does not modify the systemic immune response in peripheral lymph nodes

**Figure 5:**
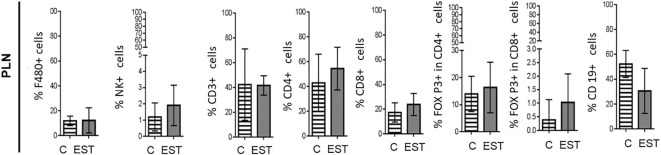
Subpopulations of cells in PLN from animals injected intratumorally with EST. Determination of the frequencies of Macrophages (F4/80+), Natural killer (NK), T Lymphocytes (CD3+), helper T Lymphocytes (CD4+), cytotoxic T lymphocytes (CD8+), Treg CD4+, Treg CD8+ and B (CD19+) lymphocytes. The bars represent the average ± SD (Vh, n = 5; TE, n= 8).

In order to evaluate the inflammation promoted by their secretion and excretion products (EST) from *T. canis* in the proximity of the tumor, we performed measurements of the subpopulations in the peripheral lymph nodes (PLN) of the animals injected intratumorally with the vehicle or EST. In none of the subpopulations measured significant differences were found between cell proportions: Macrophages (F4/80+), Natural killer (NK), T Lymphocytes (CD3+), helper T Lymphocytes (CD4+), cytotoxic T lymphocytes (CD8+), Treg CD4+, Treg CD8+ and B (CD19+) lymphocytes in the PLN (**Figure 5**).

### 3.5 Modulation of cytokine level in the spleen

**Figure 6:**
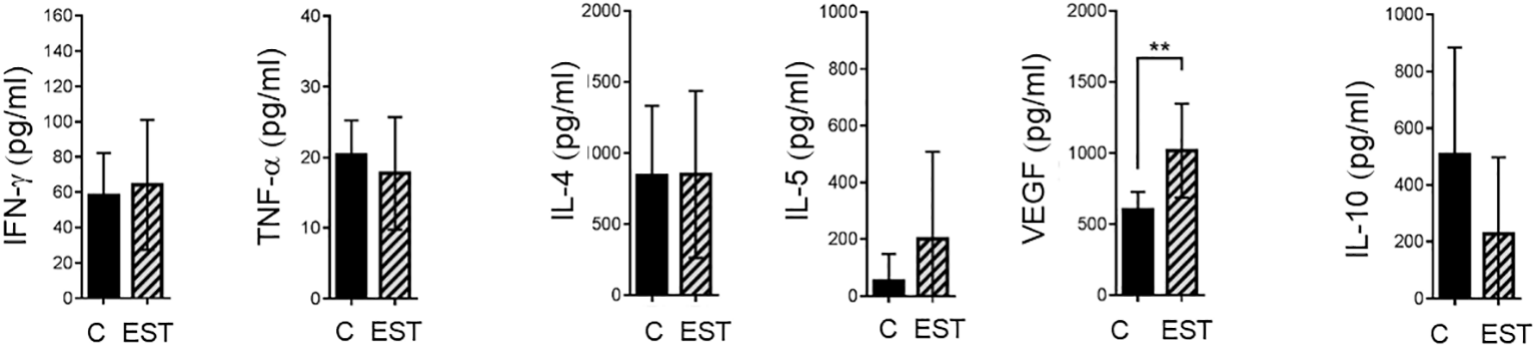
Analysis of systemic soluble factor expression in serum. Determination by sandwich ELISA of indicated cytokines: IFN-γ, TNF-α; the type 2 cytokines: IL-4, IL-5, VEGF; and the regulatory cytokine IL-10. Graphs indicate data from sera obtained from two independent experiments (4T1, n = 10; 4T1+T. EST, n= 10). Bars represent the mean ± SD of cytokine levels (pg/mL of sera). Statistical significance was calculated using a t-test (*p ≤ 0.05);.

Expression of systemic soluble factors was performed to determine whether the changes in the tumor microenvironment were reflected at the systemic level (**Figures 6**). We determined, type 1 cytokine IFN-γ, TNF-α and type 2 cytokine IL-4, the regulatory IL-5 and IL-10 in mice injected intratumorally with EST compared with vehicle (C) but showed no significant statistical differences at the systemic level (**Figure 6**). We also determined VEGF as one of the key factors that allow tumor progression and facilitate metastasis; we found in the serum an increased level of VEGF (p = 0.0030) in animals injected with EST after implantation of 4T1 cells compared with control group injected with PBS and 4T1 as well (**Figure 6**). The elevated VEGF levels in the serum were higher in animals injected with EST and 4T1 compared to animals with EST without tumor (data not shown), indicating that EST could facilitate tumor growth and metastasis.

### 3.6 Macrometastasis in the lung but no signs in liver

**Figure 7:**
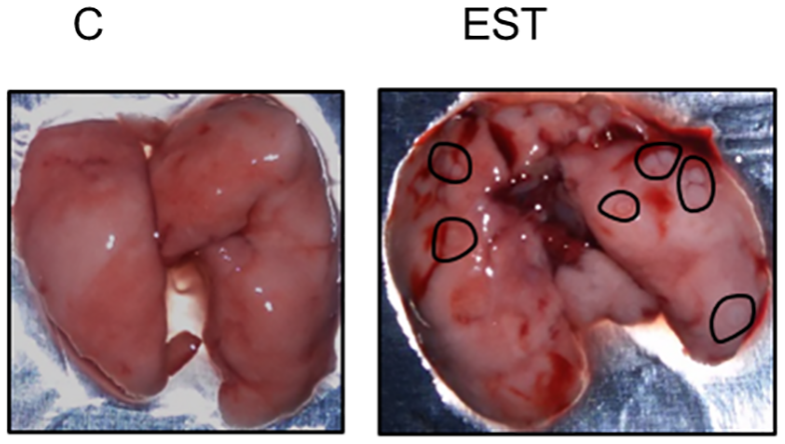
Macroscopic study of metastasis in the lungs. Representative photography of lungs from control animals (C) compared to EST+4T1 injected animal. Black circles indicate macrometastasis.

One of the physiopathologies observed in breast cancer that increases mortality is metastasis, and the main target organs are lungs. Even though the difference in the size of the tumors to which the EST were injected was not significant, we detected macroscopic lung lesions and high vasculature suggestive of higher metastasis of the animals in the EST group (**Figure 7** -right). Besides, several lesions were detected, we also observed an increased vascularity in the lungs and even areas of hemorrhages, while lungs without EST were apparently with no signs of metastasis neither area of high vasculature. The EST group without tumor showed no apparent damage in the lugs (data not shown).

Breast cancer can also metastasize to the liver. We observed no macroscopic lesions in the liver of the animals of the two groups with tumors (4T1 and 4T1+EST) (data not shown).

### 3.7 Microarchitecture of the lung tissue

**Figure 8:**
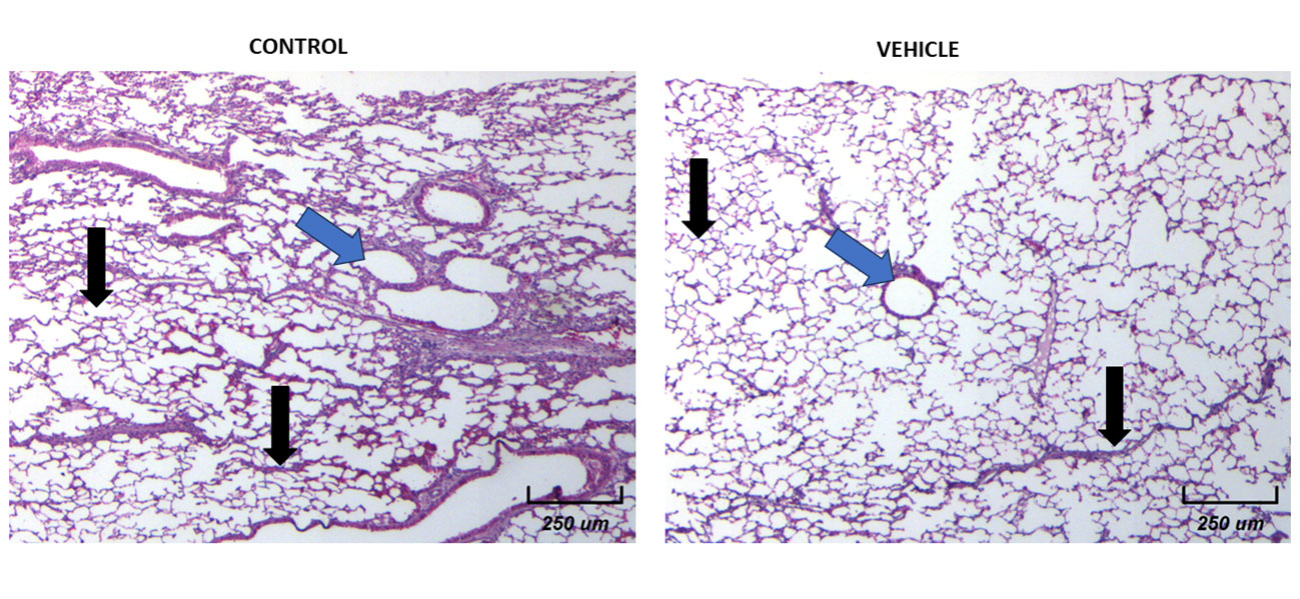
Histological examination of female mice lungs without tumors in both Control and Vehicle mice. 10X Magnification of lung tissues for both groups. There is a normal histological appearance in intact or vehicle lungs.

Then we decided to explore the histological architecture of the lungs in all experimental groups. To achieve a better view, we decided to divide the plates. **Figure 8** shows the comparison of the microarchitecture of the lungs between control and vehicle groups. We found that there is no inflammation, nor necrotic foci, and no new vascularization in any of the representative images shown (black arrows). The architecture of the lungs remains organized and with an appropriate air flow (blue arrows).

### 3.8 Micro metastasis of the lung tissue in animals implanted with 4T1 without EST

**Figure 9:**
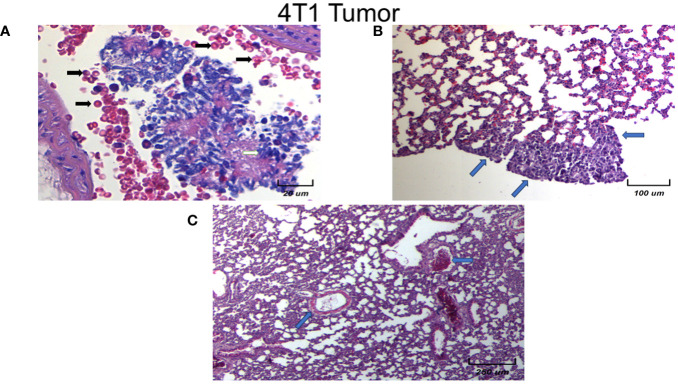
Histological examination of female mice lungs with tumors without EST. Different magnification of lung tissues of different experimental groups. There is a slight infiltration of lymphocytes. Representative images of the lungs in female mice with tumor induction at different magnifications (250×, 100×, and 20×). Only controls are shown, since vehicle treated animals behave in the same fashion. Parenchymal **(A)** and sub-pleural **(B, C)** micrometastasis in the lungs of mice orthotopically implanted at eight weeks old with tumor cell line 4T1. Representative images of the lungs of female mice with tumor induction.

Micrometastasis of control and vehicle animals is shown at the pulmonary level to analyze the morphology and cellular infiltrate. We observed several different types of micrometastasis multiple, bilateral, sharply outlined, rapidly growing, more pleomorphic, and necrotic sites in both groups (black arrows). It is important to highlight that the vehicle-treated animals with 4T1, have similar histology patterns and metastasis as the control 4T1 group. In **Figure 9** we show the histological examination of female mice normal metastasis. In general, in this experimental model of breast tumor, there is metastasis to the lungs after 21 days. The lung metastasis was generally multiple, well-circumscribed, and tended to grow rapidly (**Figure 9A**, black arrows). They appeared as multiple discrete nodules in the periphery of the lungs or as lymphangitic carcinomatosis (peribronchial and perivascular patterns via the lymphatics) (**Figure 9B**, blue arrows). Rarely appear as intra-lymphatic microscopic foci that cause pulmonary hypertension (**Figure 9C**, blue arrows). These histopathological changes were associated with decreased alveolar ventilation in mice implanted with 4T1, which presented subpleural and parenchymal metastasis.

### 3.9 Histological examination of lungs and micro metastases in animals treated with EST

**Figure 10:**
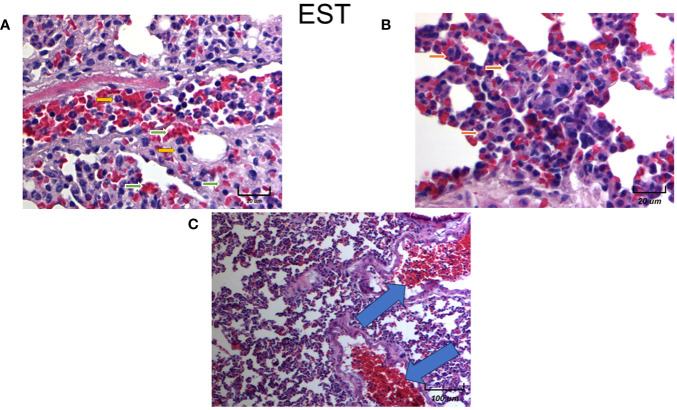
Histological examination of female mice lungs injected with EST intratumorally. Representative images of the lungs in female mice with tumor induction and treated with EST at different magnifications. Parenchymal (**A** - 250X) and sub-pleural (**B** - 250X, **C** - 20X) micrometastasis in the lungs of mice orthotopically implanted at eight weeks old with tumor cell line 4T1 and injected with EST. Representative images at indicated magnifications.

We found an increase in the inflammatory infiltrate by neutrophils of the alveolar wall and neutrophilia in intratumoral injected animals with EST (**Figure 10A** yellow arrows). Notably in mice exposed to EST (EST/4T1) the inflammatory infiltrate, tissular damage and necrotic loci were highly elevated (**Figure 10A**). The infiltration showed presence of macrophages (orange arrows), high numbers of erythrocytes (**Figure 10A** green arrows), decreased alveolar ventilation and subpleural and parenchymal metastasis (**Figure 10B** orange arrows). Clearly, the EST treated group has more micrometastasis and high immune cell infiltration compared to the control 4T1 and vehicle groups (**Figure 10C**, blue arrows). These histopathological changes showed a decreased alveolar ventilation in mice implanted with 4T1, but also showed an important loss of architectures of the lungs with remarkable subpleural and parenchymal metastasis (blue arrows).

## 4 Discussion

Cancer research has focused on a cell-centered approach, based on signaling pathways and changes in DNA (26). With the main objective of finding the “cure for cancer” and providing more personalized treatments. This type of research has yielded different advances in the cell biology and treatment of this disease, but the same attention has not been paid to other aspects of cancer development, such as its risk factors. These involve the interactions between cells, their microenvironment, and with other organisms. Within these interactions, the immune system plays a key role in tumor development that can be controlled if immunosurveillance mechanisms work properly. Both tumor cells, immune cells, and other pathogenic organisms co-evolved through interactions that may or may not favor the development of morbidities.

Tumors are very heterogeneous and consist of many different cells, including of course immune cells, in addition to the tumor cells. Interactions of all these cells results in primary tumor proliferation and, as a result, high vascularization, and metastasis. These cellular and peripheral signals are referred to as the Tumor Microenvironment (TME), consisting of immune cells, endothelial cells, adipocytes, cancer-associated fibroblasts (CAFs), and non-cellular substances such as ECM and cytokines (24). This mixture of components of TME impacts the outcome of the patient since it affects cancer initiation, progression, metastases, and treatment resistance (25). On the other hand, secretion and excretion products (EST) from *T. canis* stimulate inflammation which promotes recruitment, polarization, or differentiation of immune cells to control the infection; in addition, this inflammation could promote tumor progression by different means.

Parasites regulate the host’s immune response to promote their survival. This regulation has been documented and is mediated by the excretion and secretion products of helminths, which promote increasing levels of different immune cell populations such as Alternatively Activated Macrophages (AAM), lymphocytes, Tregs, Bregs and soluble factors produced by these cells (27). The regulation of the immune response allows the survival of helminths, but it can also favor the progression of the tumor. In the present work, we studied the effect of intratumoral injection of excretory/secretory antigens of the human parasite *Toxocara canis* (TES) on the progression of murine mammary tumors and metastasis. We used the 4T1 model because the immune system of the BALB/c mice is not compromised as in other models, which allowed the evaluation of the local (tumor) and systemic immune responses. As mentioned, helminths can survive in their hosts for long periods of time, inducing a chronic immune response. *T. canis* induces Type 2 regulatory responses and decreases the production of Type 1 cytokines (23, 28, 29).

In addition to the presence of Treg cells, helminths induce other types of cells with regulatory functions, such as Breg and Alternatively Activated Macrophages (AAM) (30). Although further characterization is needed to determine the phenotype of B Lymphocytes and F4/80+ macrophages, the increased proportions of these two populations in the spleen, coupled with a microenvironment rich in IL-4, IL-10, VEGF, and decreased levels of TNF-α and IFN-γ, suggest a regulatory phenotype of both cell types. This is supported by reports of *in vitro* studies, where macrophages from *T. canis*-infected mice were cultured to produce higher amounts of IL-10 and lower amounts of TNF-α than uninfected mice (31). There are studies where some helminths are inducers of different types of tumors (18); the coexistence of diseases caused by helminthic infections and the promotion of tumor growth has also been described (32). A model intestinal infection by the nematode *Trichuris muris* accelerated the development of intestinal adenomas in APC min/+ mice (33). In these two studies, the nematode and the tumors are in the same anatomical location.

The local and systemic increase in VEGF in ESTs has been characterized by the presence of serine proteases, which can degrade components of the extracellular matrix such as laminin and fibronectin, as well as albumin and caprine IgG (19, 34). These proteases are an important invasion mechanism for the larva. We hypothesize, that a similar mechanism could be working for the invasion and metastasis of neoplastic cells that need to penetrate through the basement membrane and remove the extracellular matrix from the tissues, carried out by proteases that degrade the components of the extracellular matrix (35). Although we did not determine the presence of ESTs in the tumor microenvironment, it has been reported that these products can be detected in the serum of infected mice (36).

Therefore, we injected ESTs directly into the tumor to observe their immunomodulatory effect against tumor formation and evaluate the tumor microenvironment and metastasis. Our results showed there was no significant increase in the size of the tumors or differences in the tumor subpopulations or in secondary lymphatic organs. This is contrary to what has been reported in other models of tumors, since the injection of antigens obtained by sonication of larval eggs of *T. canis* before the inoculation of the WEHI-164 line of fibrosarcoma, can decrease the size of these tumors (37). With intratumoral injection of EST, the increase in gross lung metastases was found to be more evident in these animals than in mice injected with the vehicle alone.

Furthermore, Hypoxia-Inducible Factor (HIF)-1 may have a role during metastasis induced by ESTs. HIF-1 is a dimeric protein complex that plays an integral role in the body’s response to low oxygen concentrations, or hypoxia. Upregulation of HIF-1α activates many crucial cancer hallmarks such as angiogenesis, glucose metabolism, cell proliferation/viability, invasion and metastasis and has a crucial role in tumor survival and progression. Thus, HIF-1 is among the primary genes which can increase vascularization in hypoxic areas such as tumors. HIF-1 transcriptional regulation over VEGF it is important to highlight. One of the most striking hallmarks of invasive cells is epithelial-to-mesenchymal transition (EMT), which is symbolized by impairment of epithelial cell-to-cell contact and the attainment of mesenchymal traits and motility. HIF-1 enhance the expression of vascular endothelial growth factor (VEGF). This is associated with increased tumor growth and metastatic spread of solid malignancies, including human breast carcinomas. It has been suggested that persistent activation of STAT3 is linked to tumor-associated angiogenesis, but underlying mechanisms remain unclear. Therefore, it is postulated that STAT3 may modulate the stability and activity of hypoxia-inducible factor-1α (HIF-1α), and in turn enhances VEGF expression.

HIF-1 is also essential for immunological responses vascularization, and anaerobic metabolism, both associated to metastasis. Furthermore, HIF-1 is increasingly studied because of its perceived therapeutic potential. Because HIF-1 allows for survival and proliferation of cancerous cells due to its angiogenic properties, its inhibition potentially could prevent cancer metastasis. So, an important follow up of this study, would be to study the role of HIF-1 in the induced ESTs metastatic process.

Perhaps a limitation of our study it is the direct injections of the ESTs antigens into the tumor, since infection, as well as ESTs, are systemic, and, they are present in circulation and in many cases in different organs. Nevertheless, neither the parasite has been demonstrated to be present inside the tumors, and, it may be more difficult that to happen. It is more feasible that ESTs may enter the tumor and, induce their immunomodulatory effect in the tumor itself, affecting the tumor immune microenvironment. However, augmented tumor growth associated with *T. canis* infection, and ESTs secretion is the result of complex interactions among the immune system, tumor cells, and the nematode larvae or their antigens. In this intricate network, the immune response must act against two different etiologies that usually occur in everyday life and in all kinds of organisms.

Another possible limitation of the present study, it is that, since no differences in the percentage of immune cells were detected in the tumor, spleen, and PLN, we did not provide some evidence about the functionality of these cells in specific, in order to explain our findings in an expanded better way. However, the indirect way was to measure system circulating cytokine levels, which we did found out differences. Furthermore, intratumoral detection of any of the angiogenic cytokines was probed in the present manuscript, which could shed more light about how metastasis it is being increased by the intratumoral treatment. We are currently working on both issues, to decipher the exact molecular mechanisms associated with increased metastasis due to ESTs treatment.

The host immune response is induced by the *T. canis* larvae excretory-secretory (TES) products, and the evasion of this response allows the nematode to survive for many years in different host tissues, which point to T cannis infection as a very important risk factor of co-morbility in other diseases, such as breast cancer.

Nevertheless, in human breast cancer patients or even in companion animals such as dogs and cats with mammary tumors, the screening of anti-T. canis antibodies to identify the infection is recommended to treat them and try to restrict the continuous promotion of a regulatory and angiogenic host immune response due to *T. canis.*

## 5 Conclusions

The results obtained in this work show the effect of the injection of excretory/secretory antigens of *Toxocara canis* (EST). First, we found no significant modulation in the size of tumors or the immune population within the tumor, spleen, or peripheral lymph nodes. Second, the tumor microenvironment and lung metastasis were modified by the presence of the EST, since VEGF were increased systemically, the vascularity in the tumor and the immune infiltration in the lung metastasis were higher. Finally, the damage of the lung architecture were evident showing that comorbidity of helminth infection and cancer can increase tumor progression and fatal outcome.

## Data availability statement

The original contributions presented in the study are included in the article/supplementary material. Further inquiries can be directed to the corresponding author.

## Ethics statement

The animal study was approved by The Institutional Committee for Care in the Use of Laboratory Animals (CICUAL) from Instituto de Investigaciones Biomèdicas, UNAM evaluated and approved the experimental procedures (permit number 2017-208). The study was conducted in accordance with the local legislation and institutional requirements.

## Author contributions

RA-F: Investigation, Methodology, Validation, Visualization, Writing – original draft. RR-M: Conceptualization, Formal analysis, Investigation, Methodology, Writing – original draft. KN-C: Funding acquisition, Investigation, Methodology, Writing – original draft, Writing – review & editing. VR-A: Conceptualization, Investigation, Methodology, Software, Writing – original draft. CG-C: Investigation, Methodology, Writing – original draft, Writing – review & editing. AP-T: Conceptualization, Investigation, Methodology, Software, Validation, Writing – original draft. RC-S: Resources, Supervision, Writing – review & editing. MG-P: Conceptualization, Funding acquisition, Investigation, Resources, Writing – original draft. JM-M: Conceptualization, Formal analysis, Funding acquisition, Resources, Supervision, Validation, Writing – original draft, Writing – review & editing.
